# New Insights in Celiac Disease

**DOI:** 10.5041/RMMJ.10073

**Published:** 2012-01-31

**Authors:** David Branski

**Affiliations:** Hadassah University Hospital, The Hebrew University Hadassah Medical School, Jerusalem, Israel; Editor-in-Chief, Journal of Pediatric Gastroenterology and Nutrition, Europe

**Keywords:** Celiac disease, gluten, probiotics, zonulin

## Abstract

Celiac disease (CD) is an autoimmune disorder occurring in genetically susceptible subjects. The incidence of CD is around 1%, and it is much more common in first-degree relatives of CD patients, 10%–18%. However, the pattern of the genetic inheritance is still obscure. Environmental factors are undoubtedly affecting the disease’s clinical presentation, time at presentation, and may have an effect on the characteristics of the disease. The clinical presentation of CD has shifted during the previous decades from the classical presentation in which the toddler suffers from diarrhea, constipation, vomiting, failure to thrive, abdominal distension, etc., to the child with a monosymptomatic presentation, such as anemia, as well as an enlarged list of extra-intestinal disorders. The diagnosis of CD is being established by symptoms consistent with CD and positive serology. The ultimate diagnosis should be made upon histological evaluation of the small bowel mucosa. The treatment of CD is a lifelong, strict gluten-free diet (GFD). Compliance with a GFD is quite difficult. Therefore, new strategies for prevention and treatment modalities other than GFD are greatly needed. Recently several promising therapeutic modalities have been developed; these include resuming traditional baking techniques. Another methodology is using probiotic-driven prolylendopeptidase. Another pathway to tackle the therapeutic option in CD is by down-regulation of the activity of zonulin—the active pump enabling gluten to enter the enterocytes. We are facing an era where other modalities beyond a GFD might allow CD patients to be able to tolerate occasionally a small amount of gluten in their diet.

Celiac disease (CD) is an autoimmune disorder occurring in genetically susceptible subjects. CD is the only autoimmune disease where the target of the immune reaction, namely gluten, has been identified.

The incidence of CD is around 1%, and it is much more common in first-degree relatives of CD patients, 10%–18%. However, the pattern of the genetic inheritance is still obscure. The proteins blamed for causing CD are the peptic-tryptic digest of gluten, namely gliadin, the wheat prolamines, and the related prolamines from rye and barley. Currently, the oat prolamines are considered safe in most but not all CD patients.^1^,^2^

## ENVIRONMENTAL FACTORS

Environmental factors are undoubtedly affecting the disease’s clinical presentation, time at presentation, and may affect the characteristics of the disease. There are claims that controlling some of the environmental factors might affect the development of CD. Several studies towards the end of the previous century demonstrated that breastfeeding reduced the incidence of developing CD. Was it a real prevention or just postponing its appearance, as was demonstrated later by Maki’s group from Finland?^3^ This group demonstrated that breastfeeding does indeed postpone the development of the disease in its classical presentation, to appear later in life with either symptoms derived from malabsorption, such as anemia or bone disorder, or as an extra-intestinal manifestation of CD, such as insulin-dependent diabetes mellitus (IDDM) and rheumatoid arthritis.

Recently, Norris et al.^4^ demonstrated that introducing small amounts of gluten to infants from 4–6 months old while still breastfeeding decreased the incidence of CD in a risk group for developing CD (HLADQ2 and/or DQ8-positive subjects).^5^

Infectious agents might have a role, at least on the timing of the presentation of CD or even on its incidence. A sequence homology between the toxic peptide of gliadin and enteric type Adenovirus was demonstrated by Kagnoff et al.^6^

Recently, Stene et al.^7^ demonstrated that exposure to two or more serotypes of Rotavirus is statistically significantly more common in CD. Adherence of bacterial agents to the small bowel intestinal mucosa was found in CD patients, but not in control subjects.^8^ Nieuwenhuizen et al.^9^ demonstrated that the virulent factor of *Candida albicans*—hyphal wall protein 1—shares similar sequence homology of amino acids with gliadin.

## PATHOGENESIS

In a celiac-susceptible subject with the specific HLADQ2 and/or DQ8, under stressful situations (such as infection, surgery, etc.), the gliadin enters the lamina propria where it is deamidated by the enzyme tissue transglutaminase (tTG) and then becomes attached to it. The resulting complex is presented to the antigen-presenting cell, T cell–HLADQ2/8, and hence starts multiple parallel responses. The most important is the TH1 response by which proinflammatory mediators such as transforming growth factor beta (TGF-β) and tumor necrosis factors gamma (TNF-γ) are secreted. The latter activates matrix methyl proteinases, which degrade the matrix, eventually culminating in destruction of enterocyte villi, characteristic of CD. The TH2 pathway will stimulate the B cells to produce specific immunoglobulins including anti-gliadin and anti-tTG antibodies.^1^

We have demonstrated elevated prostaglandin E2 and thromboxane B2 levels in the mucosa obtained from CD patients as compared with controls.^10^ Moreover, we have reported increased apoptosis in CD patients while on a gluten-containing diet, in comparison to controls.^11^

## CLINICAL PRESENTATION

The clinical presentation of CD has shifted during the previous decades from the classical presentation in which the toddler suffers from diarrhea, constipation, vomiting, failure to thrive (FTT), abdominal distension, etc., to the child with a monosymptomatic presentation, such as anemia, bone disorders, and arthritis, as well as an enlarged list of extra-intestinal disorders (Table 1).^12^

**Table 1:** Whom to screen?

**Possible “Atypical Presentation”**
Alopecia
Aphthous stomatitis
Enamel hypoplasia
Infertility
Intractable seizures
Lymphoma
Osteoporosis
Short stature
Unexplained aminotransferase elevation
Unexplained anemia
**“At-Risk” Groups (Associated Diseases)**
First-degree relatives
Insulin-dependent diabetes mellitus
Autoimmune endocrinopathies
Connective tissue disorders
Non-Hodgkin lymphoma

Adapted from Branski D, Troncone R. J Pediatr^12^ Copyright (1998), with permission from Elsevier.

## DIAGNOSIS

Who should be considered for screening for CD? Many diagnoses of CD are currently being performed following screening tests of first-degree relatives of CD patients; most of them are asymptomatic, others are diagnosed due to related disorders.

The diagnosis of CD is being established by symptoms consistent with CD, positive serology, i.e. high anti-tTG, endomysial antibodies (EMA), and elevated deamidated gliadin peptide antibodies (DGP), encompassing IgG as well as IgA antibodies.

As IgA deficiency is much more common in CD compared to the general population, the tTG and EMA, both belonging to the IgA immunoglobulin family, may be (false) negative in CD. Moreover, in young children, less than 2 years old, the incidence of false negative celiac serology is higher than later in life and should be taken into consideration while evaluating a child with suspected CD.

After demonstrating elevated celiac serology, the ultimate diagnosis should be made upon histological evaluation of the small bowel mucosa. The classical histopathologic findings are: villous atrophy, hyperplastic crypts, increased intraepithelial lymphocytes (IEL) infiltration (CD8), and increased inflammatory cells infiltration in the lamina propria, as well as increased mitotic index. Many experts are using Marsh histological criteria, in which stage 1 is just IEL infiltration and stage 3c shows total villous atrophy. One should always anticipate the desired improvement of the patient while on a strict gluten-free diet (GFD).

Recently, new modified guidelines for the diagnosis of CD have been published in the *Journal of Pediatric Gastroenterology and Nutrition*.^13^ Its main message is that under very strict conditions the diagnosis of CD may be made without performing a small intestinal biopsy. These conditions consist of a symptomatic patient, elevated celiac serology, namely elevated anti-tTG above 10-fold normal, confirmation with positive EMA test and positive HLADQ2 and/or DQ8. Of course, the introduction of a GFD should contribute to elimination of the symptoms and signs and normalization of the celiac serological markers. When these conditions are not being met, the diagnosis of CD must rely on small bowel biopsy. Moreover, the diagnosis of CD without performing a small bowel biopsy should be made by an expert in the field only.

## TREATMENT

The ultimate treatment of CD is a lifelong, strict GFD. Compliance with a GFD is quite difficult, especially among adolescents. The diet is much less tasty, quite expensive, and has social implications. The lack of adherence to GFD might affect up to two-thirds of the patients. Therefore, new strategies for prevention and treatment modalities other than a GFD are greatly needed. We discussed earlier the potential role of breastfeeding and the introduction of gluten to the diet while the infant is breastfed, as well as preventative measures against specific infections such as vaccination against Rotavirus. Nonetheless, recently several promising therapeutic modalities have been developed. These include resuming traditional baking techniques, by longer baking periods, with acidified dough. Another methodology is using probiotic-driven prolylendopeptidase, which is capable of digesting the toxic moiety of gliadin, rendering it harmless. Actually, a very recent publication by Greco et al.^14^ demonstrated that the dough baked with these prolylendopeptidases from probiotic microorganisms contained less than 20 ppm of gluten. More large-scale studies are indicated in order to demonstrate similar outcomes.

Another pathway to tackle the therapeutic option in CD is by down-regulation of the activity of zonulin—the active pump enabling gluten to enter the enterocytes.^15^

Decapeptide originates from durum grain and has been demonstrated to have a protective effect upon the small bowel mucosa of celiac patients manifesting with elevated IL-10 and decreased INF-gamma levels. The addition of this decapeptide might assist with other modalities in alleviating symptoms related to gluten consumption. Obviously, this methodology is not enough by itself to serve as a sole therapeutic modality.^16^

Certainly, various grains such as teff, buckwheat, and quinoa that do not contain containing gluten and related prolamines, and the more traditional flours from rice and potato, are safe for CD patients.

## CONCLUSION

In conclusion, we are approaching an era where other modalities beyond a GFD might allow some CD patients to be able to tolerate occasionally a small amount of gluten in their diet.

## Abbreviations:

CD: celiac disease;
EMA: endomysial antibodies;
FTT: failure to thrive;
GFD: gluten-free diet;
IEL: intraepithelial lymphocytes;
TGF: transforming growth factor;
tTG: tissue transglutaminase.
